# Alterations in right ventricular pumping in patients with atrial septal defect at rest and during dobutamine stress

**DOI:** 10.1186/1532-429X-17-S1-Q86

**Published:** 2015-02-03

**Authors:** Sigurdur S Stephensen, Katarina Steding-Ehrenborg, Ulf Thilén, Einar Heiberg, Håkan Arheden, Marcus Carlsson

**Affiliations:** 1 Department of Pediatric Cardiology, Lund University Hospital, Lund, Sweden; 2 Cardiac MR group Lund, Dept. of Clinical Physiology, Lund University, Lund, Sweden; 3 Department of Cardiology, Lund University Hospital, Lund, Sweden; 4 Dept. of Medical Imaging and Physiology, Lund University Hospital, Lund, Sweden

## Background

CMR can provide details on ventricular pumping by sub-dividing the contribution to stroke volume (SV) into longitudinal shortening and radial inward motion of the ventricular borders. Previous studies have shown that patients with volume loaded dilated right ventricles (RV) due to pulmonary regurgitation (PR) have decreased longitudinal contribution to RVSV. Patients with atrial septal defects (ASD) also have volume loaded dilated RVs but it is not known if this affects longitudinal shortening. The purpose of the study was to determine the contribution of longitudinal shortening and radial motion to SV during dobutamine stress and rest in patients with ASD, and to study the early effects of ASD closure on ventricular pumping.

## Methods

**Figure 1:**
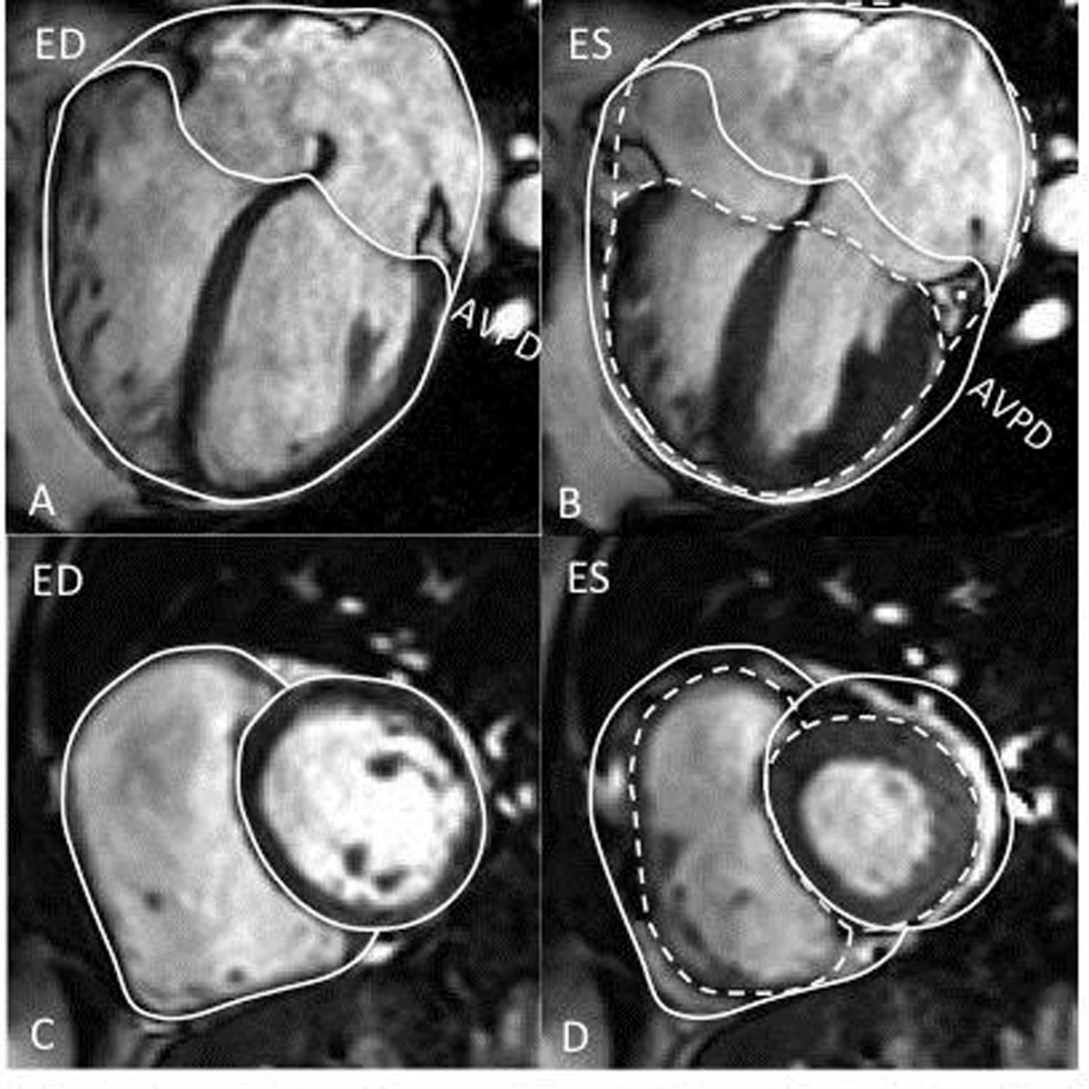
Cardiac MR images in the 4-chamber view (A and B) showing heart volume variations in a patient with ASD. The solid and the dotted white lines represent the heart contours in end-diastole (ED) and end-systole (ES), respectively. Atrioventricular plane displacement (AVPD) caused by longitudinal shortening of the ventricles is marked as a solid white line in ED and a dotted white line in ES.C and D: Short axis images of the same patient. The solid lines represent the epicardial contours of the LV and RV at ED and the dotted lines the epicardial contours at ES. This measurement was done in all short axis slices from the base to the apex. The difference in contours was used to calculate the radial contributions to LVSV and RVSV.

Eighteen patients (13 females) with ASD and 16 healthy volunteers (3 females) were imaged with CMR at rest and during a dobutamine/atropine stress protocol. Cine SSFP images were used for LV and RV volumes. Contribution of longitudinal shortening and radial motion to left ventricular (LV) SV and RVSV was measured using manual contouring of short axis images and measurement of longitudinal shortening in three long axis images (Fig 1). The septum was defined as the insertion of the RV into the LV in short axis images. The day following transcutaneous closure of the ASD, a repeated CMR at rest was performed in patients (n=15).

## Results

The left-to-right shunting ratio (QP/QS) in patients before closure was 2.2±0.8. There was no difference in the contribution of longitudinal shortening to SV in patients and controls (Table 1). During dobutamine-stress longitudinal shortening contributed less to RVSV and the contribution of radial motion increased. In patients, radial motion of the ventricular septum was towards the RV in systole, contributing to RVSV. This is in contrast to controls where septal motion was towards the LV contributing to LV stroke volume. The day after ASD-closure QP/QS was 1.3±0.2 and RV end-diastolic volume (EDV) was decreased compared to before closure (13±11%). Ventricular pumping had changed with a larger contribution of longitudinal shortening to RVSV and radial motion of the septum towards the LV.

**Table 1:** Longitudinal, lateral and septal contribution to stroke volume in patients with atrial septal defect (ASD) and healthy controls at rest and during dobutamine stress.

	ASD patients preop at rest	ASD patients during dobutamine stress	ASD patients postop at rest	Controls at rest	Controls during dobutamine stress
HR (bpm)	73±11	124±11***	63±6°°	65±10	130±12***

CO (L/min)	4.8.±0.9	8.8±2.8***	5.1±1.2	7.0±1.5	11.8±2.1***

Longitudinal contribution to LVSV (%)	63±12	56±10	56±9°	61±7	45±7***

Longitudinal contribution to RVSV (%)	75±12	61±12**	83±12°	79±9	57±10***

Lateral contribution to LVSV (%)	39±12+	46±13	38±8	31±8	46±7***

Lateral contribution to RVSV (%)	31±7	38±7*	27±10	29±7	39±10**

Septal contribution to LVSV (%)	-2±13++	-5±12	6±6°	7±4	0±6***

HR, heart rate; CO, cardiac output; LVSV, left ventricular stroke volume; RVSV, right ventricular stroke volume.

*p<0.05, **p<0.01, ***p<0.001 when comparing dobutamine stress to rest for both patients with ASD and healthy controls.

+p<0.05, ++p<0.01, when comparing patients with ASD preoperatively to healthy controls at rest.

°p<0.05, °°p<0.01, when comparing ASD patients postoperatively and preoperatively.

## Conclusions

Ventricular pumping in patients with RV dilatation caused by ASD occurs with preserved longitudinal contribution to stroke volume. This differs from previous studies of RV dilatation in patients with pulmonary regurgitation. Dobutamine stress causes similar changes in ventricular pumping in patients with ASD and controls. ASD-closure results in altered ventricular pumping mechanics as early as on day one after treatment.

## Funding

The Swedish Heart-Lung foundation.

